# A qualitative study into the experiences of occupational therapists in addressing bed positioning needs across a range of clinical settings in an area of Wales

**DOI:** 10.1177/03080226241306590

**Published:** 2025-04-21

**Authors:** Rachel Cope, Carly Reagon

**Affiliations:** Cardiff University, Cardiff, UK

**Keywords:** Occupational therapists, bed positioning, postural management

## Abstract

**Introduction:**

There is a small and low-quality body of evidence to support bed positioning as an intervention. Difficulties in developing the evidence base through experimental studies have been recognised and further research has been recommended.

**Method:**

Using a qualitative descriptive design, 13 occupational therapists were recruited from a health board in Wales, UK. Two separate focus groups (*n* = 5 and *n* = 8) were held using a semi-structured interview schedule. The data were analysed using Braun and Clarke’s six-stage approach to thematic analysis.

**Results:**

Five key themes were established. These relate to role ambiguity, variations across services, the need for training, recognising bed positioning as an individualised intervention, and effectiveness of the intervention.

**Conclusion:**

The following key findings and recommendations are outlined: (1) Bed positioning interventions are individualised. (2) The optimal timing of bed positioning assessments and interventions for inpatients is debated. (3) Multidisciplinary involvement is needed for effective implementation of bed positioning interventions. (4) Variations across services exist, causing inconsistencies in service provision. (5) More training and support in this area of practice are desired by occupational therapists. (6) More research into the clinical effectiveness of bed positioning interventions is welcomed to support clinical justification.

## Introduction

Postural distortions can occur for individuals who experience movement difficulties as a result of physical or cognitive impairments (Hill and Goldsmith, 2010). Consequently, functional difficulties and secondary health complications can arise such as comprimised skin integrity, reduced lung capacity, digestive difficulties and pain (Pope, 2007). As a result, the past two decades have seen an increased understanding in the need to support individuals in a lying position (Clayton et al., 2017). Supporting individuals in a lying position is known by numerous terms, one of which is ‘bed positioning’. Bed positioning involves the use of equipment to achieve a symmetrical and supported position of the body. However, there is a small and low-quality body of evidence to support such interventions (Humphreys et al., 2019; Robertson et al., 2018; Wynn and Wickham, 2009). As a result, further research has been recommended.

A study by Stinson et al. (2021) obtained the views of occupational therapists on bed positioning in one health care trust in Northern Ireland, with the recommendation to explore practice in other areas of the United Kingdom to build a wider picture. To date, there are no studies focusing on bed positioning practices in Wales; therefore, this study aimed to understand current practices within an area of Wales.

## Literature review

### Bed positioning assessments

Wynn and Wickham (2009) outline how occupational therapists are best placed to address bed positioning needs due to their holistic approach allowing for the consideration of the environment (bed positioning aids), person (postural needs) and occupation (functional outcomes). However, Stinson et al. (2021), highlight how occupational therapists feel less experienced in addressing bed positioning needs compared to other elements of postural management. Wynn and Wickham (2009) demonstrate some awareness of this difficulty and call for a competency framework to enable occupational therapists to work effectively in the field of postural management.

Postural distortions can develop because of movement difficulties or habitually adopted positions (Hill and Goldsmith, 2010). Congruently, research into bed positioning has been conducted with various client groups, including those with physical disabilities (Humphreys et al., 2019), learning disabilities (Robertson et al., 2018) and cognitive impairments (Fox et al., 2000), demonstrating alliance to several client groups that occupational therapists work with. Some research and guidance are condition and age-specific (Bayliss, 2020; Gericke, 2006; Gough, 2009; Hutson et al., 2021; Pountney et al., 2002), whereas others are based on postural presentation (Fox et al., 2000; Goldsmith, 2000; Innocente, 2014; Stephens and Bartley, 2020; Stinson et al., 2021). This raises questions regarding how postural management services should be targeted and compounding this, Humphreys et al. (2019) has questioned who will benefit from bed positioning.

Stinson et al. (2021) highlight how there is a lack of clinical pathway and how this is a common barrier to delivering postural care services, with some areas of the United Kingdom having pathways (Birmingham Community Healthcare, 2016), and others stating that there is not enough evidence to provide recommendations for practice (Humphreys et al., 2019). Correspondingly, ‘inconsistent provision’ (Pountney et al., 2002) and ‘fragmented services’ (Humphreys and Pountney, 2006) are terms that have been used to describe this area of practice. There is often no funding for alternative seating or lying supports and provision it is not typically coordinated to ensure that postural supports work in harmony to cover a 24-hour period (Osborne et al., 2023).

### Bed positioning interventions

Managing an individual’s posture when in a lying position considers the use of equipment to support posture (The Centre for Evidence-Based Purchasing [CEP], 2008). Equipment can vary from informal aids such as pillows and rolled up towels to equipment formally recognised and sold as postural management equipment, usually involving objects of a variety of shapes with various fillings and covers. However, decisions regarding how and what aids to use are acknowledged to be difficult for professionals, due to a lack of research evidence (Gough, 2009; Wynn and Wickham, 2009). Furthermore, there are some differences within the literature regarding how bed positioning interventions are delivered. A study by Fox et al. (2000) utilised a standardised intervention, concluding that no clinical difference was found in range of movement at knee joints, whereas a study by Stephens and Bartley (2020) utilised an individualised intervention concluding that positive effects were obtained.

### The current evidence base

There is a small and low-quality body of evidence which has explored the effectiveness of such interventions (Humphreys et al., 2019; Robertson et al., 2018; Wynn and Wickham, 2009). Existing studies contain small numbers of participants and/or involve conflicts of interests whereby equipment manufacturers have funded studies (Goldsmith, 2000; Owens and Daly, 2016).

Addressing an individual’s bed positioning needs is deemed to be effective as when asleep or inactive, the human body is more susceptible to corrective forces due to a reduction in muscle tone (Goldsmith, 2000). However, the use of bed positioning equipment is not without its critics. Gough (2009) explains how positioning equipment may interfere with sleep. Furthermore, a UK-based manufacturer of bed positioning equipment provides a checklist which outlines potential risks associated with the use of equipment, including body temperature regulation, circulation, tissue viability and comfort (Simple Stuff Works Associates, 2016).

## Methods

A qualitative descriptive design was deemed most appropriate to address the research question. Qualitative descriptive studies are considered suitable where a description of experiences and perceptions is to be obtained (Sandelowski, 2000).

Prior to data collection, approval was sought and granted from the Research and Development department affiliated with the identified health board in which the project was held. Ethical approval was granted from Cardiff University’s School Of Healthcare Sciences Research Ethics Committee in September 2021 (SREC reference: REC795).

### Data collection

Focus groups are a data collection method often associated with qualitative descriptive research (Neergaard et al., 2009; Sandelowski, 2000). Focus groups generate data from participants sharing information, experiences and points of view (Kitzinger, 1995) and were therefore deemed an appropriate data collection method to address the research question.

A purposive convenience sampling strategy was adopted due to the researcher’s employment within the health board and need to recruit participants with relevant experience (Kelly, 2010; Teddlie and Yu, 2007). The participants were contacted by emailing the head of the occupational therapy service, with the request to disseminate an introductory email regarding the study to lead therapists in each area of practice. Participant information sheets were provided to those who expressed an interest in participating in the study. Consent forms were obtained prior to participant involvement.

Fourteen participants expressed an interest to participate in the study, with 13 progressing to participating. It should be noted that out of the 13 participants, 7 were either past or current colleagues of the researcher.

Krueger and Casey (2014) explain how focus groups should involve individuals with similar characteristics. In contrast, Kitzinger (1995) explains how bringing together professionals from different areas of practice can maximise on different perspectives, which further demonstrate congruence with the aim of the study. Therefore, to strike a balance, where possible, the groups were formed with two representatives from one area of practice, and between two and four different areas of practice represented in each group (Appendix 1). The different areas of practice represented across the two groups were

1. Paediatrics (community)
2. Continuing health care (community)
3. Stroke services (inpatient)
4. Adult physical services (inpatient and community)

Virtual focus groups have been considered to meet Krueger’s (1994) criteria for focus groups by Turney and Pocknee (2005). Therefore, the groups were conducted using Microsoft Teams to create ease logistically. The groups were held in January and February 2022 for a duration of 2 hours per group.

The literature review led to the formation of questions. These questions formed a relatively structured schedule (Appendix 2). Whilst the questions were arranged in a logical order, efforts were made to ensure the style of the group discussion was conversational, to encourage discussion between participants, as suggested by Krueger and Casey (2001).

It is acknowledged that in generating questions from key issues identified within the literature, the discussions may have been restricted by presumption of what issues are important (Merton et al., 1990). Merton et al. (1990) explains how there should be a balance between addressing issues the researcher has already identified and issues not anticipated. Thus, space was provided within the groups for the discussion of unanticipated issues.

Member checking was used within the groups to achieve confirmability (Birt et al., 2016). Where a point had appeared to reach the end of its discussion, a verbal summary of the researcher’s understanding was provided to ensure it accurately represented participants experiences.

### Data analysis

The data were analysed using Braun and Clarke’s (2006) six-stage approach to thematic analysis as is it encourages the production of themes based on what is important to the research question (Braun and Clarke, 2012). The Microsoft Teams software allowed for automatic transcription, which saved time at a stage which is notoriously known for being time-consuming (McMullin, 2023). The transcript was required to be checked for accuracy as pronunciations had led to incorrect transcription in parts; however, this process was useful as it allowed for familiarisation with the data. Transcripts were then sent to the participants for confirmation that they represented their experiences. No changes were requested.

Codes were then generated inductively as, although questions had been identified to address the research objectives, to maintain congruency with the research design by obtaining participants experiences, codes were derived from the data itself, rather than in relation to each question that was asked within the group. This form of analysis is believed to encourage an unbiased development of themes by eliminating possible preconceptions of what is important in relation to the topic (Nowell et al., 2017). Most codes were generated at a semantic level. This type of code typically stays close to the data and the participants meanings (Braun and Clarke, 2012) and therefore is congruent with the qualitative descriptive design.

As the codes were reviewed, preliminary themes were entered into headings of tables, forming the third stage of thematic analysis. Themes were considered to be a pattern that captured something interesting or significant about the topic (Maguire and Delahunt, 2017), and that unified the nature of experiences into a meaningful whole (DeSantis and Ugarriza, 2000). This generated 10 themes. Under each theme, the relevant codes were placed below to enable the conversion from text to interpretation explicit (Attride-Stirling, 2001). In line with the inductive formation of codes, conscious efforts were made to not create themes in relation to the research questions, but rather in relation to the data (Nowell et al., 2017).

Themes were then reviewed and further defined, constituting stage four and five of the thematic analysis. It was at this point where some time was spent away from the data to enable consideration of the wider meaning of the themes. This led to the creation of five key themes, with up to two sub-themes in each. A mind map was created with the themes and corresponding data. This allowed for arrows to be drawn to demonstrate connections between themes which supported analysis and generated discussion within the write up, forming the final stage of thematic analysis.

The literature review was then re-read and when a point of significance was reached, the key themes mind map was referred to and connections between the findings and existing literature were made, revealing similarities and/or contrasts. These connections were annotated alongside the literature review write-up which then informed the following findings and discussion section.

## Findings and discussion

Pseudonyms are utilised throughout to preserve the anonymity of participants.

### Theme 1: A question of role

#### Subtheme: Whose role is it?

Throughout both focus groups, participants explained various reasons as to why they assess and address bed positioning needs. These included

- To promote functional positioning directly or indirectly (i.e. to free limbs to enable participation in bed or to maintain joint range to be able to sit out of bed).
- To support carers in carrying out occupations for the individual (by preventing body positions which make caring for someone difficult).
- To reduce pain/discomfort and increase comfort during sleep or when spending time in bed.
- To support emotional well-being in knowing that discomfort can cause distress and create difficulties with engaging in tasks.
- To support cognitive function in knowing how the absence of appropriate postural support can create the need to focus on maintaining ones posture and how this then impacts on one’s ability to concentrate on tasks.
- To provide experience of movement through supporting different positions for those with movement difficulties.
- To support physiological function by encouraging the typical anatomical position and shape of the body (i.e. respiratory and digestive systems).

However, despite the above, there were moments when their role was debated. This is demonstrated in the following statement:

. . .but I think there are those cases where bed positioning is for pressure care management or is arguably for a physiotherapy reason, so about maintaining joint range, or other positioning reasons that are not necessarily linked to occupation (Justine).

Bed positioning was often alluded to as a precursor to function (known as a ‘bottom-up approach’), in knowing that discomfort and poor posture will limit an individual’s ability to be occupational. However, Francesca stated:

. . .then it goes back to the question of, is it our role to do that prevention work, to talk about contractures, deformity, ‘cause if we think as a profession, we look at the impact on engagement, occupation isn’t it, so? (Francesca).

It could be argued that this thought aligns with the top-down approach to occupational therapy as appose to the bottom-up approach. This is thought-provoking in the recommendation that therapists should be aware of both approaches and select an approach that suits the needs of the individual to ensure a high quality and client-centred service (Brown and Chein, 2010).

During the discussions regarding the role of occupational therapy within this area, Sam offered a possible reason for the profession’s involvement:

It might be a historical thing because, traditionally, we’re the ones who provide the equipment [Wendy nodded]. You know that the consultant will automatically look at us to solve those sorts of issues, rather than perhaps ask other members of the MDT [multidisciplinary team].

This raises the question as to whether occupational therapists are sometimes becoming involved in this area of practice due to historical practices, rather than there being a clinical need for occupational therapy. It is argued that it is important for therapists to be clear in their role in to protect the identity of occupational therapy. However, it could be argued that this is a difficult task with limited formal guidance available, as previously identified.

#### Subtheme: The need for a multidisciplinary approach

The need for a multidisciplinary approach to postural care is outlined by Public Health England (2018b). Despite this identified need, half of the areas of practice represented within the groups (Continuing healthcare, inpatient and community adult physical services) reported having difficulties working in a multidisciplinary way, whilst the other half (inpatient stroke, children’s services and integrated adult physical services) reported multidisciplinary working to various degrees. Difficulties in working in a multidisciplinary way were considered to be due to the lack of clarity regarding who is responsible for bed positioning.

Participants particularly called for the involvement of nursing staff when considering bed positioning needs, with nursing colleagues reported as the primary referrers to occupational therapy for such needs. However, it was recognised that postural care does not appear within pre-reg nursing training (*it was acknowledged that neither does it appear within pre-reg occupational therapy training*). As a result, some therapists felt that they addressed bed positioning needs due to a lack of awareness or training within other disciplines. It is interesting to note that no research studies focusing on nursing staff and bed positioning have been identified.

Further highlighting the need for nursing involvement, Penny explained some contradictions between pressure care (which typically sits within the role of nurses) and postural management techniques:

Even though there’s stuff out there that says you don’t need to now, the NICE policies still say about turning everyone every so many hours, but this can mean people are not becoming fully relaxed to get the most benefits out of the positioning aids.

Kate detailed the multidisciplinary approach that occurs within children’s services:

So, in terms of the MDT bit, we would carry out what we call a posture screening tool so we would work alongside physios, but that’s really important anyway, for you know the impact on seating and standing frames and walking. I would just call myself a glamourous assistant to the physio. At that point I write down the notes. Then it helps inform what I’m trying to do in in bed and in seating and so then we think about what the what the risks are and why we’re going for a sleep system. We usually do the assessment jointly and then the risk assessment. It is lengthy but we’ve been proportionate. We then pull out which are the risk areas and then we then say to the paediatrician, right, this has come up in the red zone. We take photos of what we’ve tried, and we say to the paediatrician, do you feel that what we what we’re achieving in the sleep system is proportionate to the level of risk. Then the paediatrician will then say yes or no, and we often have to then arrange sleep studies as well to check in the equipment once it arrives to make sure that the child maintains their oxygen sats [saturation levels] at night. ‘cause that’s something we obviously can’t really do, and then it’s linking in with TVN’s [tissue viability nurses] at that point, so I guess that’s how the MDT works, but once you’ve got that in place, there’s nothing, as long as nothing changes then you’re just got to review that every now and again.

This process appeared unique, with therapists from other areas not undertaking such steps. Whilst there is no formal guidance regarding such processes, this point generated discussion between participants regarding whether this process should be standardised across all services, or whether there is a unique need for this within children’s services. One possible reason behind this robust process being established in children’s services may be due to research focusing more on children in this area of practice, as highlighted by Humphreys et al. (2019).

### Theme 2: Variations across services

The earlier described protocol for the assessment, issue and use of bed positioning aids followed by children’s services was considered valuable by some participants; however, it was alluded to within both groups that following the protocol can be a time-consuming process, with some therapists calling for it to be simplified. Justine provided her thoughts on such a process:

I think I see lots of strengths in that kind of approach. Kate and Julie, you both did refer to how lengthy that is, and I wonder if that’s always proportionate because I understand that some bed positioning needs might not be complex. I guess that’s where I get a little bit concerned about the documentation and the process being proportionate to need. For example, if we suggest a pillow under an arm ‘cause they have a hemiplegia, and you now have to complete a five-page document to enable you to do that I would be concerned about the impact of that on practice [Kate and Julie nodding].

Penny offered an insight into her experiences of the addressment of bed positioning needs whilst working within a mental health setting:

In older adult mental health, nobody knew anything, no one was interested [about bed positioning]. It’s interesting because you walk past and there would be a lot of patients, more than you’d expect, kind of lying there clinging onto the bed rails and you kind of know why they’re doing it, but absolutely no one else has got any other ideas

Penny’s statement mirrors the findings of Pountney et al. (2002), that services are fragmented. Intensifying this, Tracey went on to explain how management of bed positioning does not appear to be present in settings where there is a focus on ‘discharge needs’, and whilst she could appreciate that bed positioning needs may not be a priority at the time of discharge, she demonstrated the following concern:

It comes down to the clinician knowing what services and resources are out there that they can tap into upon discharge, and if they don’t know, they go and seek appropriate advice from others to support them in trying to provide postural equipment if it’s needed. But then there is the need to train those who are providing the care in the community. How confident are they?.

Wendy provided the following statement:

For me as well, it all depends how often you need to do these assessments. I’ve done two of these assessments in five years, so I’m not going to be confident and competent in doing them because I don’t have to do them very often.

Wendy’s statement echoes the findings of Stinson et al.’s (2021) study in which some therapists felt lower levels of confidence in the area of bed positioning because they engaged with it less frequently.

There was a consistent message from participants working within hospital settings regarding limited access to formal positioning aids. Tracey Stated:

. . . we’ve not got access to resources on the acute ward, so we just make do with the bits and pieces that we’ve got on the unit, which is only bed sheets, blankets and pillows and towels and things.

Several participants expressed their feelings that this difficulty was due to funding issues. Sam stated the following:

It’s difficult enough to get a specialist chair on the ward, let alone something like sleep system that is very personalised to that individual patient

These findings mirror that of the Public Health England (2018b) report, in that there are difficulties in accessing postural management equipment, with lack of funding being one of the most common reasons. It is questioned whether the small and low-quality body of evidence to support the intervention (Humphreys et al., 2019; Robertson et al., 2018; Wynn and Wickham, 2009) may serve as a barrier for accessing such resources.

Whilst therapists in hospital settings reported difficulties in obtaining formal positioning aids, Tracey identified the following challenge she encounters on the acute stroke ward:

. . . . they’re too early on in their journey for those kits to be provided on a long-term basis.

For this reason, assessment of need and requests for formal equipment were reported to occur at the end of a patient’s inpatient stay, but therapists explained how they feel this seems ‘back to front’ (Sam). They felt this created difficulty in meeting needs in a timely manner and in having enough time to educate caregivers or staff who will implement the bed positioning aids long term (Justine). To combat this, Wendy presented the idea of having access to an in-house equipment store where a variety of aids could be stored, to create a ‘try before you buy’ system. This could provide increased assurances that what was eventually purchased for the individual was appropriate for their long-term needs and would provide caregivers an opportunity to become familiar and confident with using the equipment.

Sally, who worked within continuing health care services provided her experience:

We can gain access to other services or pieces of equipment, do assessments with the reps [equipment representatives]. It’s quite nice as we have a lot of flexibility to be able to really look at this stuff.

This demonstrates the positives of having access to such equipment, but highlights the variation across services within one health board.

### Theme 3: Training

#### Subtheme: Occupational therapists’ confidence and competence

Across both focus groups, several participants (Sam, Justine, Francesca and Michelle) explained that they had not received training in relation to postural management or bed positioning specifically during their pre-registration training. Sam stated the following:

You go and do it, with very little training or support. What about our competency levels to actually assess these people? Patients or children or whoever, for this equipment. With manual handling we have to reach a certain competency to be able to use certain equipment, but nobody’s assessed me to see if I’m competent in assessing somebody for a sleep system.

This mirrors the findings of Stinson et al. (2021) that occupational therapists feel least knowledgeable about bed positioning compared to other forms of postural management and compounds the recommendation for specialist postural management training within the occupational therapy profession (Osborne et al., 2023).

Sam felt that it would be beneficial to have a minimum standard of competency within this area of practice, echoing Wynn and Wickham’s (2009) call for a competency framework to enable occupational therapists to work effectively in the field of postural management. Arguably, such a competency framework, could play a role in supporting therapists’ confidence in their skills.

Contrastingly, Fiona who worked in stroke services provided the following insight:

I’ve not had training in postural management, bed positioning either. But what we have in stroke is training on optimal positioning of bodies. I wouldn’t expect a band 5 or somebody who hasn’t been in stroke [services] to just go and issue a bed positioning aid and know exactly what they were doing, but we as seniors know what the optimal positioning of upper limbs, lower limbs, trunk etc in the bed is.

This raises the question as to whether specific bed positioning training is required, or whether there are existing learning opportunities that generate skills transferrable to this area of practice.

Tracey offered the following perspective:

I think the only way you get to know is by experiencing it. If your colleagues, professional role models, and practice settings engage in and value this area of practice, then this naturally passes on to you as a clinician.

This demonstrates a connection between experience, knowledge and skills, but raises a possible issue in that if bed positioning does not exist in all areas (Public Health England 2018b), skill development could be inconsistent.

Sally and Francesca explained their engagement with self-directed learning to improve their knowledge and skills within this area of practice. However, Sally explained that she feels that whilst she has the opportunity to spend time on such activities in her current setting, her colleagues in acute settings may be restricted. This highlights a further variation in services in the ability of professionals to engage in self-directed learning in different areas.

#### Subtheme: The need for upstream work

Concerns were raised across both focus groups regarding other professionals and informal/formal caregivers’ ability to recognise the need to make a referral to their service for the assessment of bed positioning needs. Michelle provided the following insight:

I think definitely in nursing homes, although it’s not spoken about, they think that contractures are part of getting old. That’s why the education is huge, to say that it is preventative. But once it gets to that point to try and reverse that, it’s too late. Sometimes we need to be getting involved much sooner, and I think that’s where our frustration comes from sometimes.

This is congruent with the findings of Castle et al. (2014), that multidisciplinary team members were lacking awareness of why individuals should be referred to postural care services. Castle et al. (2014) recommended that a comprehensive training pack should be designed and delivered to multidisciplinary team members to raise their awareness.

On discussing other’s ability to know when to make referrals, Francesca explained how she feels it can be difficult for those who have regular contact with individuals who may need postural care, to recognise changes in their presentation.

Participants discussed how their recommendations are disseminated to formal or informal caregivers most proximal to the individual to deliver the intervention daily. Kate, who works within children’s services, explained how she disseminates positioning plans and trains school staff who are ‘fantastic’ at carrying out the interventions. Conversely, Sam reported that she finds it difficult to train community staff when working on an adult acute ward, as she completes a follow-up visit post discharge to train one caregiver with the agreement that they will train other involved caregivers; however, Sam feels this dilutes the treatment. This raises questions as to whether there is a most effective way of disseminating such interventions.

### Theme 4: An individualised intervention

#### Subtheme: Who is bed positioning for?

Across both groups, there was a unanimous opinion that bed positioning needs should not be determined by diagnosis, but rather physical presentation. Sam offered the following opinion:

I don’t think you can categorise or group them, you have to assess to see if there is a problem and if so, can you help to relieve it. Whilst I work in neurology, I wouldn’t say that it only affects neurological patients. That would be naive of me to say so.

Adding to this picture is the statement from NHS Education for Scotland (2017), that changes in body shape can even begin in people who live sedentary lifestyles. This it throws into question the necessity of diagnostic specific guidelines and research that currently exists (NICE, 2019; Pountney et al., 2002; Public Health England, 2018a).

Participants elaborated on the presentations they encounter that trigger them to consider bed positioning needs. Kate explained how in children’s services, they are aware of an increased need for postural management for children with neurological deficits around their teenage years due to the impact growth can have on muscle tone and therefore posture. Justine explained how if someone cannot alter their own position, this would trigger her to consider the need for bed-positioning intervention. This corresponds with the consideration that reduced mobility is a key risk factor in requiring postural management by NHS Education for Scotland (2017). Hayley explained how if she has a client who has specialist seating needs, this triggers her to consider postural needs in bed. This reflects the concept that bed positioning is part of 24-hour postural management (Stinson et al., 2021). Fiona explained that characteristics such as abnormal tone, spasticity, altered position of limbs and the existence of contractures would trigger her to consider bed positioning. Hayley explained how she would consider the need for bed positioning for individuals with musculoskeletal conditions as this may mean their ability to move is affected. Consideration of musculoskeletal conditions has not been encountered within the existing literature on bed positioning despite the key risk factor of decreased mobility.

The variety of presentations described above arguably demonstrates how extensive the need for bed positioning assessments and interventions could be.

#### Subtheme: What therapists use

Across both focus groups, all but three participants explained their use of informal aids such as rolled up towels and/or blankets to support an individual’s position in bed. This was often met with statements such as ‘making do’ with informal aids, informal aids not being a ‘long term or sustainable’ option, and ‘pillows and towels are never going to work wonders’. This suggests that some participants do not feel informal aids are sufficient.

Furthermore, Fiona explained how if contractures are developing, she considers a formal sleep system. This suggests that some formal systems are considered more robust.

Contrastingly, Justine, who works in a community setting where her colleagues report no issues in accessing formal positioning aids, explained her belief that formal aids are not always needed and that informal aids can meet the need in some cases. Whilst it is known that formal and informal aids can be used to support an individual’s bed positioning needs (CEP, 2008; Pope, 2007), to date, there is no collective understanding regarding the reasons for such decisions.

Tracey and Francesca explained how all products have advantages and disadvantages, and how these will differ depending on the individual’s needs. Justine offered more insight into the consideration she makes:

I question, do they have difficulty regulating their temperature, they do become agitated during the night, are their carers experienced with bed positioning aids, do they have huge continence issues. There could be a list as long as your arm of reasons why you would or wouldn’t prescribe something.

Whilst some reasoning for therapists’ decisions of what to use has been provided, more specific details around this would be useful to understand for practice. Adding to this question, where specific aids have been named within existing research literature (Goldsmith, 2000; Stephens and Bartley, 2020), reasons for such choices are not provided. An NHS buyers guide, providing technical, operational and economic considerations that should be made in relation to several manufactures (CEP, 2008), is now outdated, with a number of current equipment manufacturers not included.

### Theme 5: Effectiveness

It was suggested that bed positioning interventions can be effective through the following statements:

It’s noticeable that those older adults who hadn’t had that kind of care as children were much more dysfunctional, uncomfortable, and in painful positions than those younger adults that are coming through. So, for me that was quite a good way to see that it really is effective. It does make a difference (Penny).

I think we could all probably remember a time where we used to see quite a lot of people with a really classic, hemiplegic gait [all participants nodded] and really high tone arm walking down the street with a stick. You do still see them, but it’s as if there’s not as many, and I think that comes down to posture, positioning, normal movement, alignment, managing tone, maybe not just within the bed setting that we’re talking about now, but that will contribute to that definitely (Tracey).

Fiona explained how in using positioning aids, their patient’s range of knee extension had improved to the point where they were now able to sit in a chair. This experience contrasts with the findings of Fox et al. (2000), that positioning aids to support knee contractures did not result in an ‘important clinical difference’.

The following statements were made in relation to bed positioning interventions:

I’ve had some nurses say, actually we’ve not had to give as much pain relief recently, I’ve noticed their demeanour is a lot more settled, they don’t seem as agitated in the bed (Fiona).

Sometimes children can tell you they’re comfortable and they can sleep through the night (Julie).

Improvements in sleep mirror the findings of Stephens and Bartley (2020), and contrast with reduction in sleep quality being identified as a possible negative consequence of bed positioning by Gough (2009).

Whilst none of the participants reported negative outcomes from the use of bed positioning equipment, which contrasts with the findings of Goldsmith (2000), Fox et al. (2000) and Stephens and Bartley (2020), Kate did express how for some individuals, postural distortion is inevitable. This is an interesting perspective considering a statement by Public Health England (2018a), within a postural care guidance document, that postural distortion is not inevitable and with the right equipment and positioning technique, body shape can be protected.

#### Subtheme: Generating the evidence base

The below quotes illustrate the difficulties therapists experience in justifying their practices which highlight the need to further develop the evidence base.

It’s hard when you’ve got nothing to back you up. When there is very limited evidence and very limited information out there and all you can say is well, I’ve watched another person do this and it worked really well. That doesn’t really carry much, does it? It’s difficult to pinpoint actual pieces of research that have been done that is going to support you on the ground. We’ve got such a of lack of evidence, lack of research, lack of pathways but lots of qualitative you know conversations and experiences that we’ve all had (Michelle).

I think it’s difficult ‘cause some of the positioning kits can be quite expensive, so if you’re asking for an outlay of money and you haven’t got anything to support you on it other than a good hunch from your experience that it’s gonna work, it’s tricky. I think there’s difficulties with outcome measures as well. To get a specific outcome measure looking just at bed positioning, well yeah, that would be interesting, helpful, but I’m not sure how. ‘cause that’s the thing with bed positioning, the outcome could be due to multiple factors, you know is there bed position better because they’re in less pain but are there in less pain because their position is better. I mean, it’s the chicken and the egg’ (Tracey).

Reps will say that they’ve got this evidence, that evidence, and you look at it and it’s not helpful in taking anything to panel for authorisation of funding. It’s really biased. It’s in a small group of a handful of people, yeah, not helpful (Michelle).

It’s really hard then to put something so individual into formalised guidance to help support people that are less experienced and need that support at the beginning, to then in turn generate the evidence base. So, it’s like a cycle (Penny).

Therapists described additional factors that have an impact on the implementation of a bed positioning plan. They alluded to the challenges this creates in optimising the outcomes of bed positioning interventions, which in turn creates difficulties in demonstrating the effectiveness of the intervention.

Justine highlighted how pressure care needs are often met in bed by a particular material of mattress, ranging from foam to air flow. However, bed positioning aids do not have this same variation and so there is a risk of pressure damage from bed positioning aids. Yet, no pressure damage from bed positioning aids was reported across either group, which is a contrast to the findings of Fox et al. (2000) where reddening on knees was found. Nevertheless, some therapists reported that pressure care needs are often ranked higher on the list of priorities than postural management; therefore, the latter is unable to be addressed to its full potential, if at all.

Kate stated the following:

It’s a challenge when you’re trying to marry up postural control versus sleep ‘cause the two don’t always go together. We’ll see loads of kids, where they want to sleep on their tummies, or their sides and we want to put them on their back ‘cause we know that that’s better for their long-term postural management, but they can’t sleep on their backs.

This corresponds with the research by Goldsmith (2000) in which some children had difficulty in achieving an acceptable sleeping position, acting as a barrier to the effective use of bed positioning equipment.

Justine highlighted that bed positioning aids can become lost during the laundering process in communal care environments. It was unclear whether this resulted in an impact on the delivery of the intervention. However, if replacement aids are not sourced in a timely manner, it could be argued that this could have an impact on the outcomes of such interventions.

With occupational therapists delegating the ongoing implementation of bed positioning interventions to formal and informal caregivers, Sally raised a concern for the continuity of the implementation of the intervention:

There’s only so much you can do, and I suppose when you’re having to leave your intervention in in the duty of care of others [Michelle and Penny nodded] and hope that they will take note and they will monitor, and they will review and highlight.

It could be argued that if this statement is true, that the outcomes of bed positioning aids would be hindered, and in turn, the generation of its evidence base.

Participants discussed how often, individuals are receiving several interventions including postural management at other times of the day, medication or physical therapy, which may explain the outcomes aimed to be achieved by bed positioning interventions. This highlighted the difficulties in being certain that it was bed positioning aids alone that had influenced an outcome.

## Conclusion

This study is one of the first to explore bed positioning from the perspective of occupational therapists within Wales. It has shed light on how services are delivered and some clinical reasoning behind therapists’ decisions, whilst adding information to some key questions raised within the literature.

In response to the questions regarding who benefits from bed positioning, participants in this study indicated that they do not consider an individual’s diagnosis when determining the need for bed positioning, but rather their individual physical characteristics. Whilst therapists within this study provided some examples of the physical characteristics they consider, further understanding of such characteristics would be useful to guide practice.

What aids therapists use and how they use them when addressing bed positioning needs was considered to be individualised by participants and some reasons for their choices have been provided. This contrasts with a study where a standardised treatment method was utilised and no benefit was concluded (Fox et al., 2000), further suggesting that an individualised approach to this treatment is appropriate. A new insight was provided regarding the timings of bed positioning assessments for inpatients, with uncertainty as to whether they are best to occur and the beginning or end of an inpatient’s stay. Further exploration of such decisions would be useful to aid practice.

Whilst the participants saw an alliance between their role and addressing bed positioning needs, the need for a multidisciplinary approach was advocated unanimously. Whilst a multidisciplinary approach to this area of practice is supported by Public Health England (2018b), half the areas of practice reported difficulties in working multidisciplinary, whilst the other half reported multidisciplinary working to various degrees. It would be useful to further explore the fit of bed positioning with the role of occupational therapy and other professionals such as nurses.

Variations in services were reported by participants, mirroring findings within the literature. Reasons for this included varying awareness and therefore following of an in-house protocol with mixed views on its helpfulness, logistical differences such as access to resources, and the clinical need not arising frequently within caseloads which create uncertainty for therapists in their ability to meet the clinical need.

In line with the findings of Stinson et al. (2021), several participants questioned their competencies within this area of practice and called for formal training and a competency framework. The education and training of other professionals and caregivers was deemed to be essential to optimise the timeliness and successfulness of the intervention.

Participants reflected on cases where the intervention had been effective, but there was the opinion of one participant that sometimes, postural distortion is inevitable. This generates doubt over the effectiveness of bed positioning and conflicts with a statement by Public Health England (2018a). Further research into the effectiveness of the intervention was called for, to support therapists’ clinical justification.

### Strengths and limitations

The rich and diverse discussions held during the data collection as a result of grouping occupational therapists from various practice areas, to engage in a two hourly discussion are considered a strength of this study, allowing valuable insight into occupational therapists’ practices. An indirect benefit also occurred in that within both groups, participants expressed the value they found in engaging with the focus groups as a way of listening to each other’s experiences, obtaining knowledge and support from others, and sharing resources. Participants felt that this is something that should occur more frequently.

Whilst the value of the researchers’ experiences in completing this study is appreciated and attempts were made to be reflexive, there is awareness of the difficulties in entirely removing this influence during data collection and analysis. In the later, continuous reference to original data assisted in minimising bias.

It is acknowledged that where there is a pre-existing relationship between the researcher and the participant/s, individuals may volunteer to participate because of their desire to help rather than altruistically wishing to contribute to the research study (McConnell-Henry et al., 2014). To compound the possibility of this occurrence the researcher’s employment history is within physical services, and there was no representation from the mental health and learning disabilities division. Whilst pre-existing relationships may have impacted upon the sample characteristics, this cannot be concluded, as factors such as not wishing to participate or not meeting the inclusion criteria may explain the lack of representation from other areas of practice.

This study may be transferrable to other UK, public sector occupational therapy services; however, caution is needed due to the relatively small sample size. The absence of data regarding participants years of experience and/ or post qualification training also presents some challenges as individual therapists’ knowledge, skills and therefore experiences may vary depending on this. In retrospect, it would have been a useful to collect such data for greater transferability, and for a possible deeper understanding of the reported experiences.

Furthermore, it is acknowledged that there may be local variations in the way services are structured and the availability of resources, which could impact upon occupational therapists’ experiences. However, the reported lack of national clinical guidance and research evidence could be argued to be a barrier in supporting occupational therapists within this area of practice and are likely to have an impact upon the design and delivery of postural care services across the UK.

The inclusion of ‘current clinical setting’ data is deemed a useful tool to support the understanding of which specific services these findings may be applicable to.

**Key findings**

- Bed positioning interventions are individualised.
- The optimal timing of bed positioning assessments and interventions for inpatients is debated.
- Multidisciplinary involvement is needed for effective implementation of bed positioning interventions.
- Variations across services exist, causing inconsistencies in service provision.
- More training and support in this area of practice are desired by occupational therapists.
- More research into the clinical effectiveness of bed positioning interventions is welcomed to support clinical justification.

**What the study has added**

This study has provided an insight into occupational therapist’s bed positioning practices in an area of Wales.

It has emphasised the necessity to further explore the fit of bed positioning with occupational therapy and other professionals such as nurses.

It has highlighted the need to consider the timing of bed positioning assessments for patients in hospital.

It has also raised questions regarding the usefulness of a local protocol, despite a lack of clinical pathway being considered a barrier to delivering postural care services.

## Appendix 1

Focus group demographics.

**Table:**

Focus group 1	
Pseudonym	Current clinical setting
Tracey	Inpatient stroke services
Michelle	Continuing health care, community
Sally	Continuing health care, community
Penny	Adult physical integrated services
Francesca	Children’s services, community

**Table:**

Focus group 2	
Hayley	Continuing health care, community
Sam	Inpatient stroke services
Justine	Continuing health care, community
Fiona	Inpatient stroke services
Julie	Children’s services, community
Kate	Children’s services, community
Wendy	Community adult physical services
Jackie	Inpatient adult physical services

## Appendix 2

### Questioning schedule

1. Who do we think requires bed positioning?
2. What sorts of things do you think about before addressing bed positioning needs?
3. Pick up on/probe where barriers and/ or supporting factors are alluded to. If these are not, ask ‘do we feel there are things that make addressing bed positioning needs easy or difficult?’
4. What does implementing bed positioning interventions look like in practice?
5. Do we have any ideas about the effectiveness of bed positioning interventions?
